# Trigeminal neuropathy presenting secondary to SARS-CoV-2 infection

**DOI:** 10.1097/PR9.0000000000001103

**Published:** 2023-10-17

**Authors:** Francis O'Neill, Gianfranco De Stefano, Mike Pridgeon, Deepti Bhargava, Anne Marshall, Andrew Marshall, Bernhard Frank

**Affiliations:** a Institute of Life Course and Medical Sciences and the Pain Research Institute, University of Liverpool and Liverpool University Hospital NHS Foundation Trust, Liverpool, United Kingdom; b Department of Human Neuroscience, Sapienza University of Rome, Rome, Italy; c Department of Clinical Neurophysiology, The Walton Centre NHS Foundation Trust, Liverpool, United Kingdom; d Department of Neurosurgery, The Walton Centre NHS Foundation Trust, Liverpool, United Kingdom

**Keywords:** Neuropathic, Pain, Facial pain

## Abstract

**Introduction:**

A 58-year-old woman presented to a multidisciplinary facial pain clinic in October 2021 complaining of a constant pain in the right side of her face since contracting coronavirus SARS-CoV-2 18 months earlier. The pain extending from the right temple down to her right cheek extraorally and including the maxillary teeth and right side of tongue intraorally. This was accompanied by anosmia, diplopia on lateral gaze, and dizziness.

**Methods:**

Clinical examination was supplemented with several neurophysiological tests to confirm the diagnosis including an MRI brain scan, quantitative sensory testing, electrophysiological blink reflex testing, corneal confocal microscopy, and pain and short-form anxiety and depression questionnaires.

**Results:**

Quantitative sensory testing showed unilateral loss of perception in thermal and mechanical sensibility and bilateral hyperalgesia indicating central sensitization. Bilateral corneal confocal microscopy showed an abnormally reduced corneal nerve fibre length on the right side. MRI, blink reflex, and masseter inhibitory testing findings were normal.

**Conclusion:**

This case study is the first case of trigeminal neuropathy related to SARS-CoV-2 infection reported in the literature. It also discusses the successful management of the patient's trigeminal neuropathic pain.

## Abstract 2

This case study describes a patient presenting with trigeminal neuropathy associated with SARS-CoV-2 infection and their management.

## 1. Introduction

Infection with SARS-CoV-2 leads to widespread nervous system involvement.^12^ Multiple cranial neuropathies secondary to COVID-19 have been described in the literature.^3^ These include optic nerve, oculomotor nerve, abducens nerve, and facial nerve palsies. More specifically, high levels of SARS-CoV-2 were found in the trigeminal ganglion after autopsy in COVID-19–infected patients.^7^

Trigeminal neuralgia presentation related to COVID-19 has also been reported, both as a sole symptom of COVID-19 and after vaccination against SARS-CoV-2.^5,8^ Trigeminal neuropathy has been reported in a single patient 1 month after being pain free following a microvascular decompression for trigeminal neuralgia but subsequently receiving tozinameran vaccination against SARS-CoV-2.^9^ However, here we present the first report of trigeminal neuropathic pain occurring as a result of COVID-19 in a patient naive of vaccination along with several other cranial neuropathies.

## 2. Case study

A 58-year-old right-handed woman presented to a multidisciplinary facial pain clinic in October 2021 complaining of a constant pain in the right side of her face, extending from the right temple down to her right cheek, lip, and side of nose extraorally and including the maxillary teeth and right side of tongue intraorally. The character of the pain consisted of throbbing, burning, and tingling sensations. These pains had been present since contracting coronavirus SARS-CoV-2 in March 2020 during her role as a hospital nurse manager. The coronavirus infection was confirmed with a positive serum antibody test result. The pain she described had never remitted since starting. It had a minimum severity of 7 of 10 on a numerical rating scale but could increase to 10 of 10 at times. Pain was usually worst around 2 am to 5 am in the morning which disturbed her sleep. There were no autonomic or migrainous features and she reported a subjective feeling of numbness on the right side.

During the first few weeks of infection, she lost her sense of smell and taste and neither of these senses had returned at initial presentation or by the end of patient review. She also complained of dizziness and intermittent diplopia. The patient was fit and well with no underlying medical issues and was taking no medication. There was no history of previous facial trauma, recent dental treatment, or other infections such as herpes virus infection. Owing to the pain, she had taken an extended period of sickness leave from her job and was currently not working.

The patient reported that the use of capsaicin cream, ice, and laying on the affected side helped to partially reduce the pain severity.

She had consulted her general medical practitioner for other aches in her muscles, wrists, and elbows and was being investigate for polymyalgia rheumatica. A tapering dose of steroids helped reduce discomfort in her wrists and elbows but not her facial pain.

On examination, the range of head and neck movements was within normal limits. Cranial nerve testing revealed anosmia and diplopia on lateral gaze to the left and right with reduced activity of the lateral rectus muscles (worse on the right) indicating an abducens nerve deficit. Reduced sensation to touch, pinprick, warm, and cold on bedside testing in the left V1 and right V1-3 distributions of the trigeminal nerve including numbness over the right maxillary mucosa intraorally. There was no evidence of cheek biting. Hearing was intact, but the symptoms of dizziness and some balance issues indicated a possible deficit in the vestibular system. Second, 3rd, 4th, 7th, 9th, 10th, 11th, and 12th cranial nerves were intact and normal.

We discussed the findings and suggested that these are in keeping with a diagnosis of trigeminal neuropathic pain with concomitant sixth and eighth cranial nerve neuropathies. The temporal correlation to the episode of infection with coronavirus is strongly suggestive of a causative factor.

## 3. Methods

Four neurological tests were conducted.

(1) An MRI of the brain including the trigeminal nerve was acquired with gadolinium contrast.
(2) Quantitative sensory testing was conducted using a Medoc TSA II thermosensory stimulator (Medoc Ltd, Ramat Yishay, Israel) performed on the right and left cheek (V2 distribution) only, following the standardized protocol of the German Research Network on Neuropathic Pain.^11^ Data were collected and transformed to Z scores based on normal ranges.
(3) Electrophysiological blink reflex and masseter inhibitory response testing were conducted according to established protocols.^1^ The masseter inhibitory reflex latency and blink reflex R1 and R2 components and habituation were compared bilaterally and to normal reference ranges.^1^
(4) Corneal confocal microscopy assessment of small nerve fibre measurements HRT III RCM (Heidelberg Engineering). Metrics measured were corneal nerve fibre density (CNFD), corneal nerve branch density (CNBD), and corneal nerve fibre length (CNFL). Cutoff points were used based on ACCMetrics, Petropoulos et al.^10^:

Abnormal CNFD ≤ 14.7 fibres/mm^2^, sens/spec of 0.76/0.72, and OR of 11.0.

Abnormal CNFL ≤ 14.6 mm/mm^2^, sens/spec of 0.77/0.74, and OR of 12.9.

## 4. Results of neurological testing

### 4.1. Magnetic resonance imaging

The MRI of brain with contrast excluded a space-occupying lesion and did not show enhancement on any of the cranial nerves.

### 4.2. Quantitative sensory testing

**Figure 1.**
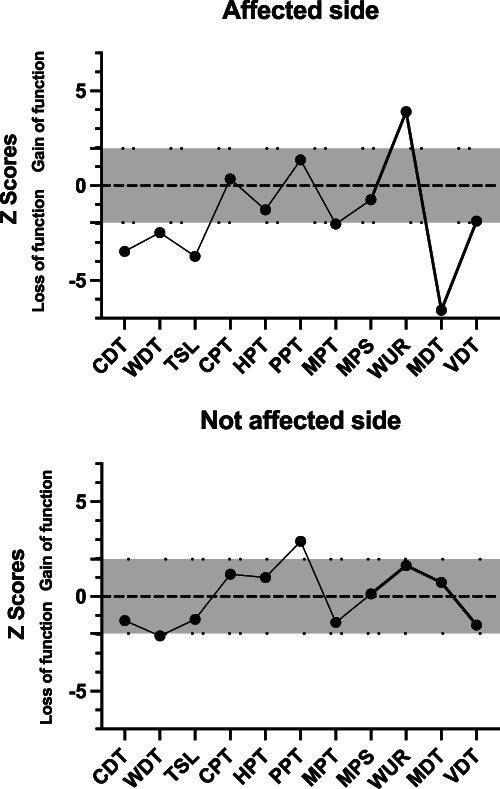
Z scores of quantitative sensory testing showing unilateral loss of perception in thermal and mechanical sensibility (CDT, WDT, TSL, and MDT) on the affected side. Also shown is a mechanical hyperalgesia (WUR) indicating central sensitization. The nonaffected side shows testing thresholds within the normal range (gray area between dotted lines). CDT, cold detection threshold; CPT, cold pain threshold; HPT, heat pain threshold; MDT, mechanical detection threshold; MPS, mechanical pain sensitivity; MPT, mechanical pain threshold; PPT, pressure pain threshold; TSL, thermal sensory limen; VDT, vibration detection threshold; WDT, warm detection threshold; WUR, wind-up ratio.

Quantitative sensory testing (QST) demonstrated a reduction in thermal and mechanical sensibility including cold detection threshold, warm detection threshold, thermal sensory limen, mechanical pain threshold, and mechanical detection threshold on the affected side and mechanical hyperalgesia indicating central sensitization. The nonaffected side shows testing thresholds within the normal range—gray area between dotted lines shown on Figure 1. Using the deterministic algorithm proposed by Vollert et al.,^13^ the sensory phenotype of the affected side could be allocated to sensory loss (Table 1).

**Table 1:** Probabilities of sensory loss (SL), thermal hyperalgesia (TH), mechanical hyperalgesia (MH), and healthy (H) sensory profiles.

	SL probability	TH probability	MH probability	H probability
Right face	0,700033564	0,417503111	0,597442924	0,402410844
Left face	0,562812478	0,649795872	0,670047021	0,562435767

### 4.3. Trigeminal reflex testing

Neither masseter inhibitory reflex nor supraorbital blink reflex testing showed any abnormalities.

### 4.4. Corneal confocal microscopy

**Figure 2.**
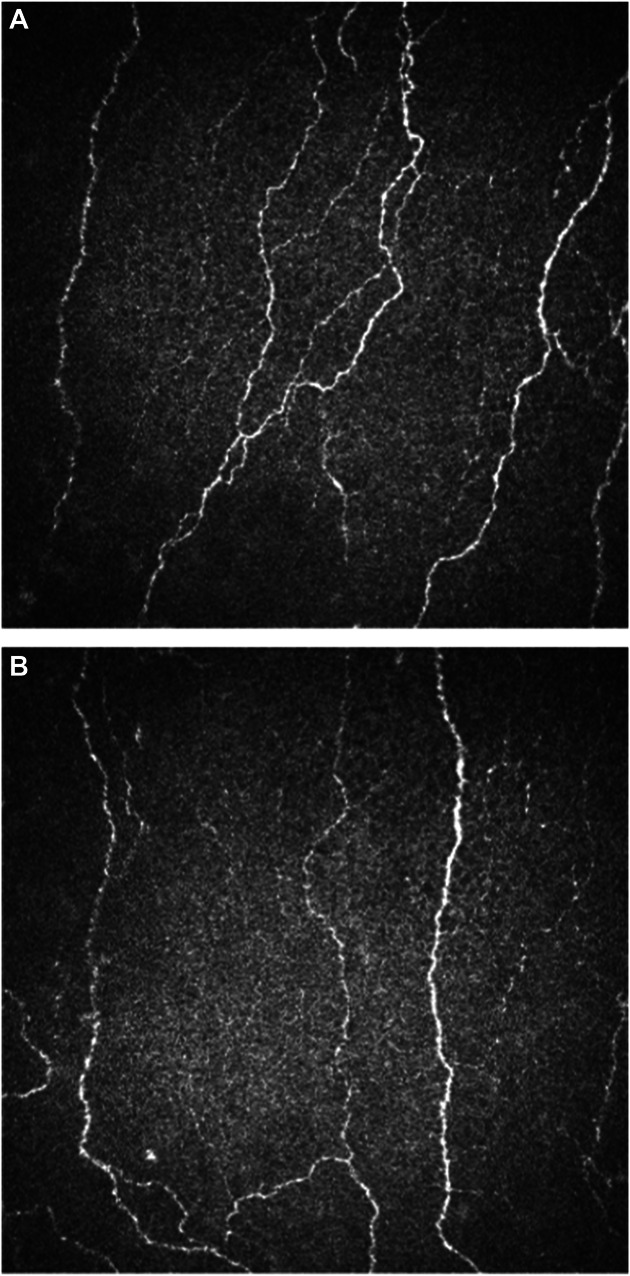
Corneal confocal micrographs showing nerve fibres in the cornea of the left and right eyes. (A) Unaffected side with normal nerve fibre density and branching and length. (B) Affected side with reduced nerve fibre length and branch density.

Corneal confocal microscopy was performed on both left and right corneas (images shown in Fig. 2). Results of the image analysis showed an abnormally reduced CNFL on the right side. Although the CNBD was within normal limits on both sides, the branch density on the affected right side was reduced to less than half that of the corresponding unaffected left side (results provided in Table 2).

**Table 2:** Results for corneal confocal microscopy.

CCM	CNFD	CNBD	CNFL
Right	20.3112	21.8736	13.4631
Left	24.9984	43.7472	14.6691

CCM, corneal confocal microscopy; CNFD, corneal nerve fibre density; CNBD, corneal nerve branch density; CNFL, corneal nerve fibre length.

### 4.5. Pain and short-form anxiety and depression questionnaires

Before appointment, the patient was asked to complete both Graded Chronic Pain Scale version 2.0^14^ and PHQ-4 questionnaires.^6^ The patient scored as grade IV-severely limited on the 30-day Graded Chronic Pain Scale with high scores for character pain intensity, pain-related interference, and disability days. She also scored 6 of a total 12 points on the PHQ-4 indicating a moderate distress.

## 5. Management

Medications previously tried by the patient had included amitriptyline, nortriptyline, carbamazepine, gabapentin, pregabalin, propranolol, co-codamol, and prednisolone. None of these medications had helped her symptoms.

The plan formulated at the end of the initial consultation was a trial of duloxetine titrating the dose up to 60 mg daily if required. If unsuccessful in reducing her pain, then intradermal botulinum toxin A injections were considered.

On orthoptic assessment in April 2022, her abducens nerve palsy had resolved and she no longer experienced diplopia and no further sixth nerve weakness clinically. At the same appointment, the patient reported no benefit from the trial of duloxetine and having already failed multiple antineuropathic pain medications, she was considered for a trial of intradermal botulinum toxin A (Allergan, Marlow, United Kingdom). For the first series of botulinum toxin A injections, 150 units was given intradermally over all 3 branches of the trigeminal nerve on the affected right-hand side only. Three months after this, the patient reported a partial response, and therefore, a second series of injections with a further total of 150 units botulinum toxin A was given. At 6-month follow-up, the patient reported a greater than 50% reduction in her pain scores and that the pain in the maxillary and mandibular branches had gone. Therefore, a third course of botulinum toxin A injections was given at a reduced dose of 50 units to the remaining affected ophthalmic branch area only.

On subsequent follow-up in June 2023, the patient was again asked to complete both Graded Chronic Pain Scale version 2.0 and PHQ-4 questionnaires. The patient scored as grade 0-no limitation or disability on the 30-day Graded Chronic Pain Scale and scored 0 of 12 points on the PHQ-4 indicating no distress. She reported that she was pain free and scored 0 of 10 on the numerical rating scale. She had also returned to work.

## 6. Discussion

Here, we describe a patient who presented with a trigeminal neuropathy with a close temporal relationship with antibody-proven SARS-CoV-2 infection. The patient's symptoms together with clinical signs and results of quantitative sensory testing are in keeping with a diagnosis of trigeminal neuropathic pain. The finding of normal reflexes does not contradict this diagnosis.^4^

Quantitative sensory testing identified in this patient a marked objective sensory loss involving sensory modalities mediated by both small (cold detection threshold, warm detection threshold, and mechanical pain threshold) and large (mechanical detection threshold) afferents, indicating a nonselective trigeminal damage. The sparing of vibration detection threshold, also mediated by large fibres, may be due to transmission of vibrations to the contralateral unaffected side through skull bones, which may have hindered a unilateral sensory loss.

The finding of a right trigeminal neuropathy together with the presence of other cranial neuropathies which occurred at the same time as the SARS-CoV-2 infection would suggest that the causative agent is likely to be SARS-CoV-2 and follows other reports of multiple cranial nerve deficits associated with this virus.

SARS-CoV-2 virus could infect the central and peripheral nervous systems by several potential means, eg, hematogenous spread through either the blood–brain barrier or the blood–cerebrospinal fluid barrier.^15^ Furthermore, SARS-CoV-2 attaches to ACE2 receptors and these have been found to be expressed on neurons and glial cells.^2^ Retrograde transport of virus could occur along cranial nerves, and viral RNA has been detected in olfactory sensory neurons and the trigeminal ganglion.^2^ This finding of viral RNA in the trigeminal ganglion is direct evidence of trigeminal involvement. Finally, the clinical presentation as multiple cranial neuropathies supports the hypothesis of a direct viral infection spreading among contiguous nerves. Unilateral presentations are commonly seen in other viral infections, for eg, herpes zoster. Conversely, a bilateral symmetric presentation would have been more likely in the case of postinfective dysimmune mechanisms, such as those seen in post-COVID-19 Guillain–Barré syndrome.^3^

We believe this to be the first case of trigeminal neuropathy related to SARS-CoV-2 infection reported in the literature. Moreover, this is the first described case in which corneal confocal microscopy is applied to trigeminal neuropathic pain, showing a marked asymmetry in corneal nerve branch density. This technique could bring a unique contribution to the diagnosis of trigeminal neuropathic pain, but further studies are needed.

## Disclosures

The authors have no conflict of interest to declare.

## References

[1] Aramideh M Ongerboer DeVisser BW. Brainstem reflexes: electrodiagnostic techniques, physiology, normative data, and clinical applications. Muscle Nerve 2002;26:14–30. 12115945 10.1002/mus.10120

[2] Bauer L Laksono BM de Vrij FMS Kushner SA Harschnitz O van Riel D. The neuroinvasiveness, neurotropism, and neurovirulence of SARS-CoV-2. Trends Neurosci 2022;45:358–68. 35279295 10.1016/j.tins.2022.02.006 PMC8890977

[3] Costello F Dalakas MC. Cranial neuropathies and COVID-19: neurotropism and autoimmunity. Neurology 2020;95:195–6. 32487714 10.1212/WNL.0000000000009921

[4] Jääskeläinen SK Teerijoki-Oksa T Forssell H. Neurophysiologic and quantitative sensory testing in the diagnosis of trigeminal neuropathy and neuropathic pain. PAIN 2005;117:349–57. 16153774 10.1016/j.pain.2005.06.028

[5] Kaya A Kaya SY. A case of trigeminal neuralgia developing after a COVID-19 vaccination. J Neurovirol 2022;28:181–2. 34870807 10.1007/s13365-021-01030-7 PMC8647511

[6] Kroenke K Spitzer RL Williams JBW Lowe B. An ultra-brief screening scale for anxiety and depression: the PHQ-4. Psychosomatics 2009;50:613–21. 19996233 10.1176/appi.psy.50.6.613

[7] Meinhardt J Radke J Dittmayer C Franz J Thomas C Mothes R Laue M Schneider J Brünink S Greuel S Lehmann M Hassan O Aschman T Schumann E Chua RL Conrad C Eils R Stenzel W Windgassen M Rößler L Goebel HH Gelderblom HR Martin H Nitsche A Schulz-Schaeffer WJ Hakroush S Winkler MS Tampe B Scheibe F Körtvélyessy P Reinhold D Siegmund B Kühl AA Elezkurtaj S Horst D Oesterhelweg L Tsokos M Ingold-Heppner B Stadelmann C Drosten C Corman VM Radbruch H Heppner FL. Olfactory transmucosal SARS-CoV-2 invasion as a port of central nervous system entry in individuals with COVID-19. Nat Neurosci 2021;24:168–75. 33257876 10.1038/s41593-020-00758-5

[8] Molina-Gil J González-Fernández L García-Cabo C. Trigeminal neuralgia as the sole neurological manifestation of COVID-19: a case report. Headache 2021;61:560–2. 33749854 10.1111/head.14075 PMC8251254

[9] Onoda K Sashida R Fujiwara R Wakamiya T Michiwaki Y Tanaka T Shimoji K Suehiro E Yamane F Kawashima M Matsuno A. Trigeminal neuropathy after tozinameran vaccination against COVID-19 in postmicrovascular decompression for trigeminal neuralgia: illustrative case. J Neurosurg Case Lessons 2022;3:CASE22101. 36303493 10.3171/CASE22101 PMC9379720

[10] Petropoulos IN Alam U Fadavi H Marshall A Asghar O Dabbah MA Chen X Graham J Ponirakis G Boulton AJ Tavakoli M Malik RA. Rapid automated diagnosis of diabetic peripheral neuropathy with in vivo corneal confocal microscopy. Invest Ophthalmol Vis Sci 2014;55:2071–8. 24569580 10.1167/iovs.13-13787 PMC3979234

[11] Rolke R Magerl W Campbell KA Schalber C Caspari S Birklein F Treede RD. Quantitative sensory testing: a comprehensive protocol for clinical trials. Eur J Pain 2006;10:77–88. 16291301 10.1016/j.ejpain.2005.02.003

[12] Satarker S Nampoothiri M. Involvement of the nervous system in COVID-19: the bell should toll in the brain. Life Sci 2020;262:118568. 33035589 10.1016/j.lfs.2020.118568 PMC7537730

[13] Vollert J Maier C Attal N Bennett DLH Bouhassira D Enax-Krumova EK Finnerup NB Freynhagen R Gierthmühlen J Haanpää M Hansson P Hüllemann P Jensen TS Magerl W Ramirez JD Rice ASC Schuh-Hofer S Segerdahl M Serra J Shillo PR Sindrup S Tesfaye S Themistocleous AC Tölle TR Treede RD Baron R. Stratifying patients with peripheral neuropathic pain based on sensory profiles: algorithm and sample size recommendations. PAIN 2017;158:1446–55. 28595241 10.1097/j.pain.0000000000000935 PMC5515640

[14] Von Korff M Ormel J Keefe FJ Dworkin SF. Grading the severity of chronic pain. PAIN 1992;50:133–9. 1408309 10.1016/0304-3959(92)90154-4

[15] Zubair AS McAlpine LS Gardin T Farhadian S Kuruvilla DE Spudich S. Neuropathogenesis and neurologic manifestations of the coronaviruses in the age of coronavirus disease 2019: a review. JAMA Neurol 2020;77:1018–27. 32469387 10.1001/jamaneurol.2020.2065 PMC7484225

